# Tumor Necrosis Factor-*α* T-857C (rs1799724) Polymorphism and Risk of Cancers: A Meta-Analysis

**DOI:** 10.1155/2016/4580323

**Published:** 2016-12-27

**Authors:** Ping Wang, June Wang, Mingxia Yu, Zhiqiang Li

**Affiliations:** ^1^Department of Clinical Laboratory and Center for Gene Diagnosis, Zhongnan Hospital of Wuhan University, Wuhan 430071, China; ^2^Guangdong Provincial Key Laboratory of Medical Molecular Diagnostics, Guangdong Medical University, Dongguan 523808, China; ^3^Department of Neurosurgery, Zhongnan Hospital of Wuhan University, Wuhan 430071, China

## Abstract

*Objectives*. To investigate the potential association of tumor necrosis factor-*α* T-857C polymorphism with susceptibility to the five common malignant tumors.* Materials and Methods*. A comprehensive search of PubMed/Medline, Embase, and Web of Science databases was performed up to November 2015. Pooled odds ratios (ORs) and 95% confidence intervals (95% CIs) were calculated to assess the strength of the association. Subgroup analysis, heterogeneity analyses, and publication bias were also texted in the meta-analysis.* Results*. A total of twenty-two publications involving 5215 cases and 6755 controls were recruited. Overall, the meta-analysis revealed an increased risk between the TNF-*α* T-857C polymorphism and gastric cancer susceptibility in T versus C model, heterozygote genetic model, and dominant genetic model. An increased risk between the TNF-*α* T-857C polymorphism and hepatocellular cancer susceptibility in homozygote genetic model and recessive genetic model was also found. No significant association was found between the TNF-*α* T-857C polymorphism and colorectal cancer, cervical cancer, and prostate cancer.* Conclusions*. Our meta-analyses suggest that TNF-*α* T-857C polymorphism may be associated with increased risk of gastric cancer and hepatocellular cancer development. Therefore, the TNF-*α* T-857C polymorphism could be considered as one possible risk factor of gastric cancer and hepatocellular cancer according to our study.

## 1. Introduction

Cancer has been a disease which endangers human physical and psychosocial wellbeing, causing a significant public health and economic burden all over the world. Cancer is a multifactorial disease, and the etiology of these cancers is extremely complex. In order to understand its pathology, numerous susceptibility genes and external environmental factors appear to be considered.

Chronic inflammation has long been associated with the development of cancer. Recent evidences have reignited the interest of cancer researchers in the exciting concept of an association between chronic inflammation and cancer. Rather than protecting against cancer, growing evidence indicates that TNF-*α* can promote the development of cancer [1]. Previously our study also found that targeting TNF-a suppressed breast cancer growth and TNF-*α* monoclonal antibody exerted effectively antitumor activity [2], which further supported this assertion.

The length of TNF gene is 12 kilobases (kb) and it is located on the short arm of chromosome 6 (p21.1–p21.3) [3]. As transcription of TNF-*α* is regulated under genetic control, recent studies have shown that its promoter polymorphisms at TNF-*α* G-238A (rs361525), TNF-*α* G-308A (rs1800629), TNF-*α* T-857C (rs1799724), and TNF-*α* T-1031C (rs1799964) positions could regulate TNF-a production, thus affecting the risk of cancers [4–7]. Therefore, genetic polymorphisms of TNF-*α* gene have been supposed as candidate risk factors of cancer.

There are a large number of studies on the association between TNF-*α* G-308A (rs1800629), TNF-*α* G-238A (rs361525), and cancers [8, 9]; TNF-*α* G-308A (rs1800629) and TNF-*α* G-238A (rs361525) have been successfully identified as risk factors of cancer. Molecular epidemiological research suggests that TNF-*α* T-857C (rs1799724) polymorphisms may be associated with an increased risk of cancers [10–13], but results remain controversial. TNF-*α* T-857C is a C to T transition in the promoter at position −857, and previous data have shown that TNF-*α* T-857C allele T increases the transcription of TNF-*α* [14–16]. Therefore, TNF-*α* T-857C polymorphism may be associated with cancer risk and represents candidate risk marker of cancers. To explore a more precise estimation of the relationship between TNF-*α* T-857C polymorphism and cancers, we performed a meta-analysis.

## 2. Materials and Methods

### 2.1. Study Selection

To identify eligible studies published before November 2015, we applied a systematic literature search strategy to the following electronic databases: PubMed/Medline, Embase, and Web of Science. We used the following keywords and subject headings in combination to identifying relevant articles in electronic databases: (tumor necrosis factor alpha OR TNF-*α* OR 857 C/T OR rs1799724) AND (polymorphism OR variant OR genotype OR mutation) AND (cancer OR carcinoma OR neoplasm). Duplicate articles were manually filtered using the “find duplicate function” of EndNote X7. Two of the authors reviewed results of each of the database searches to make sure that published papers were not missed.

### 2.2. Inclusion and Exclusion Criteria

The following inclusion criteria were used for the literature selection: (1) genotype distributions of both cases and controls were available; (2) the studies might be cohort or case-control studies; (3) articles about TNF-*α* T-857C polymorphism and cancer risk; (4) sufficient published data for estimating an odds ratio (OR) with 95% confidence interval (CI); (5) when several publications were available for the same study group, we retained the most recent one for analysis. We excluded publications as follows: (1) based on pedigree data that were excluded; (2) retrospective or cross sectional studies; (3) nonoriginal research (reviews, editorials, or commentaries), abstracts, unpublished studies, and duplicated studies; and (4) studies on animals.

### 2.3. Data Extraction

Two authors (Ping Wang and June Wang) independently extracted characteristics of studies and resolved any uncertainty through discussion. If these two authors could not reach a consensus, a third author was consulted to resolve the dispute and a final majority decision was made. After excluding the overlap studies and including the additional ones, this meta-analysis covered a total of 22 articles on TNF-*α* T-857C polymorphism. From each identified article, we extracted the first author's name, publication year, country, ethnicity, type of disease, sample size, source of control, genotyping method, and Hardy-Weinberg equilibrium (HWE) for controls.

### 2.4. Statistical Analysis

The Hardy-Weinberg equilibrium (HWE) in the controls was tested by the Chi-square test for goodness of fit. The following contrasts for the associations between TNF-*α* T-857C polymorphism and the cancers mentioned above were evaluated: T allele versus C allele, homozygote comparison (TT versus CC), heterozygote comparison (TC versus CC), and recessive (TT versus TC + CC) and dominant (TT + TC versus CC) genetic model, respectively. The strength of association between TNF-*α* T-857C polymorphism and cancer risk was assessed using the pooled odds ratio (OR) with 95% confidence intervals (CIs). If the number of included studies was applicable, subgroup analysis was performed based on HWE status of controls, ethnicity, source of control, and genotyping method. The heterogeneity of the data was quantified using Chi-square statistics. Heterogeneity among studies was considered significant when *P* < 0.1 or *I*^2^ > 50%. If there was significant heterogeneity among studies, the random effects model (DerSimonian and Laird) was used; otherwise, the fixed-effects model (Mantel and Haenszel) was acceptable. We plotted Begg's funnel plot to examine the underlying publication bias. We conducted sensitivity analysis by deleting each included study in turn to evaluate the overall robustness of the study's results. All analyses were conducted using Review manager 5.3 and Stata 12.0. All the *P* values were two-sided.

## 3. Results

### 3.1. Study Selection and Characteristic

**Figure 1:**
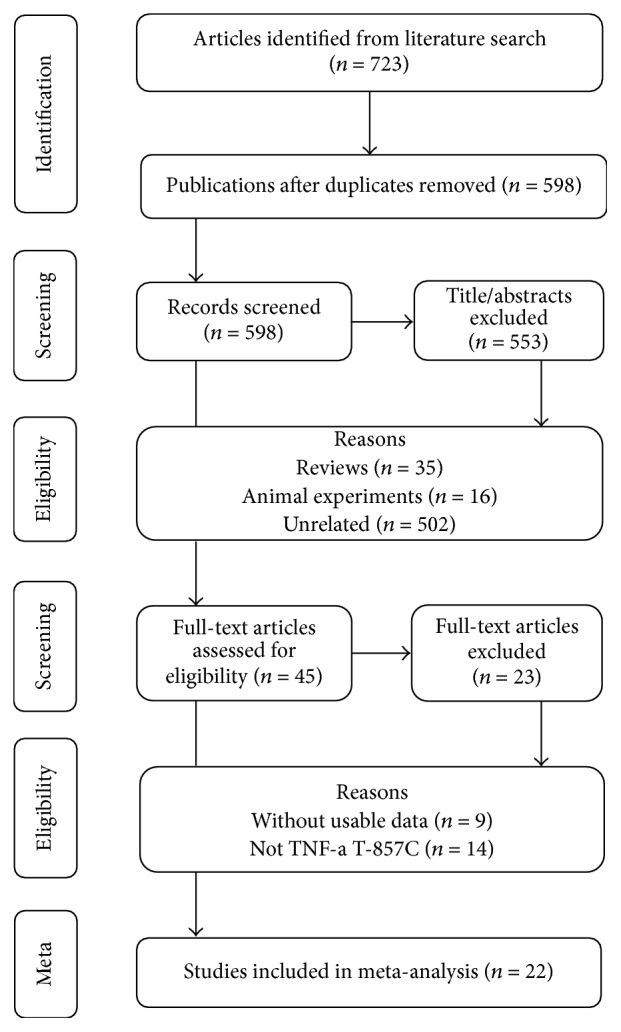
Flow diagram of the study selection process.

There were 723 papers relevant to the search words. The flowchart of selection of studies and reasons for exclusion is presented in Figure 1. Finally, a total of 22 studies were included in this meta-analysis. All those 22 studies were reported in English. The details of included studies were shown in Table 1. In the initial search, 723 articles were retrieved, and 553 studies were excluded after reading titles and abstracts. 5215 cases and 6755 healthy controls were included in this study. There are 9 studies for gastric cancer [17–25], 4 studies for colorectal cancer [26–29], 4 studies for hepatocellular cancer [30–33], 3 studies for cervical cancer [34–36], and 2 studies for prostate cancer [37, 38]. Among those 22 studies, 10 studies were from Caucasian populations, and 10 studies were from Asian populations, while 2 studies were from African populations. The sample size of cases ranged from 84 to 1139, while the sample size ranged from 55 to 1378 in the controls. Cases were histological diagnosed in almost all studies. Most studies used healthy subjects as controls. All studies indicated that the distribution of genotypes in the controls was consistent with Hardy-Weinberg equilibrium, except 3 studies [19, 25, 33]. Several genotyping methods were used, including TaqMan, PCR-RFLP, and PCR.

### 3.2. Quantitative Synthesis

The summary results of meta-analysis of the association between the TNF-*α* T-857C polymorphism and cancer risk are displayed in Table 2.

#### 3.2.1. Association between the TNF-*α* T-857C Polymorphism and Gastric Cancer Risk

**Figure 2:**
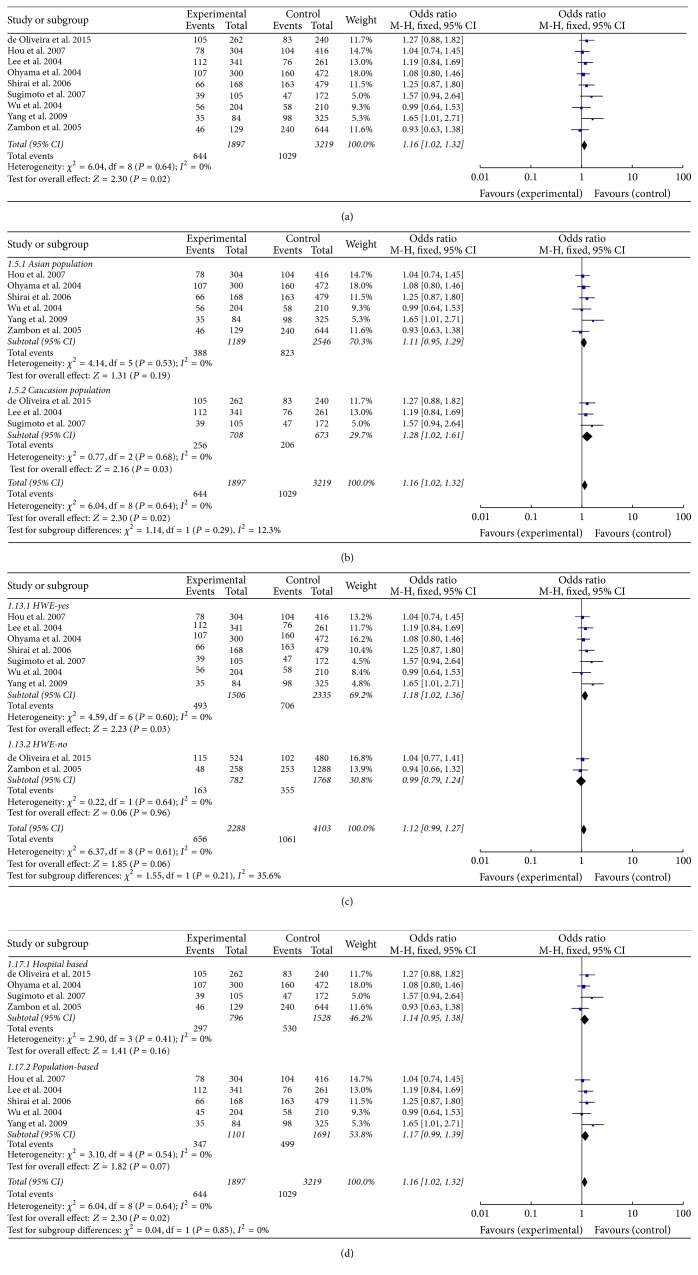
Calculated OR and 95% CIs for the associations TNF-*α* T-857C polymorphism and gastric cancer risk in the TT + TC versus CC model ((a) for overall populations; (b) for ethnicity subgroup; (c) based on HWE for controls; (d) for control sources subgroup).

**Figure 3:**
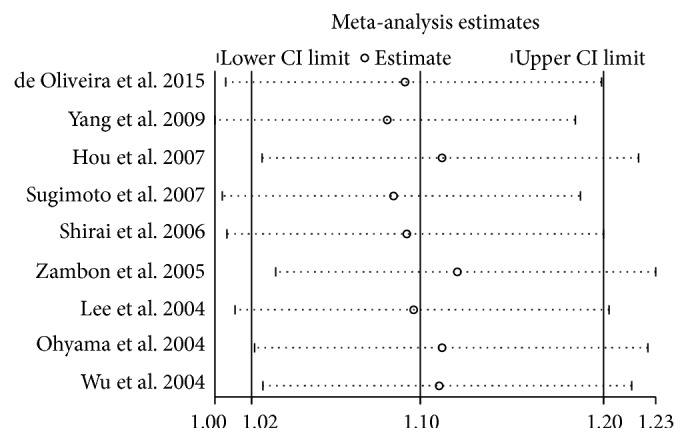
The plot of sensitivity analysis for evaluating the association between TNF-*α* T-857C polymorphism and gastric cancer risk in the TT + TC versus CC model.

A total of 9 relevant studies, consisting of 1897 patients and 3219 controls, were examined for the association between the TNF-*α* T-857C polymorphism and gastric cancer risk. In all subjects, meta-analysis showed an increased risk between the TNF-*α* T-857C polymorphism and gastric cancer susceptibility in three genetic models (T versus C: OR = 1.12, 95% CI = 1.01–1.25, *P* = 0.04, *I*^2^ = 0%, fixed-effects model; TC versus CC: OR = 1.16, 95% CI = 1.02–1.33, *P* = 0.02, *I*^2^ = 0%, fixed-effects model; TT + TC versus CC: OR = 1.16, 95% CI = 1.02–1.32, *P* = 0.02, *I*^2^ = 0%, fixed-effects model, Figure 2). Further stratified analyses based on ethnic subgroups revealed similar results in Asian populations for the T versus C model, TC versus CC model, and the dominant model. The sensitivity analysis showed the results without substantive change (Figure 3 for TT + TC versus CC model).

#### 3.2.2. Association between the TNF-*α* T-857C Polymorphism and Hepatocellular Cancer Risk

**Figure 4:**
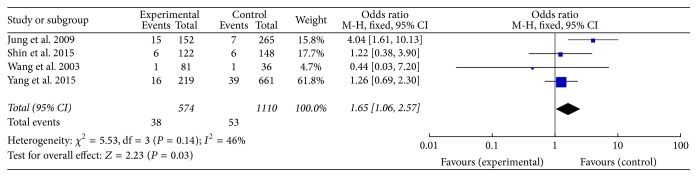
Calculated OR and 95% CIs for the associations TNF-*α* T-857C polymorphism and hepatocellular cancer risk in the TT versus CC model.

Four studies consisting of 807 cases and 1510 controls were included in this analysis. All these four studies are based on Asian population. A significant increase in hepatocellular cancer risk was observed in the Asian population in two gene models (TT versus CC: OR = 1.65, 95% CI = 1.06–2.57, *P* = 0.03, *I*^2^ = 46%, fixed-effects model; TT versus TC + CC: OR = 1.61, 95% CI = 1.04–2.49, *P* = 0.03, *I*^2^ = 37%, fixed-effects model, Figure 4). Furthermore, analysis by excluding studies with control inconsistent with HWE showed elevated cancer risk in homozygote comparison and recessive genetic model (TT versus CC: OR = 2.29, 95% CI = 1.16–4.51, *P* = 0.02, *I*^2^ = 46%, fixed-effects model; TT versus TC + CC: OR = 2.21, 95% CI = 1.12–4.93, *P* = 0.02, *I*^2^ = 37%, fixed-effects model). Sensitivity analyses were also conducted, and no conspicuous change of the pooled ORs was detected.

#### 3.2.3. Association between the TNF-*α* T-857C Polymorphism and Colorectal Cancer Risk

**Figure 5:**
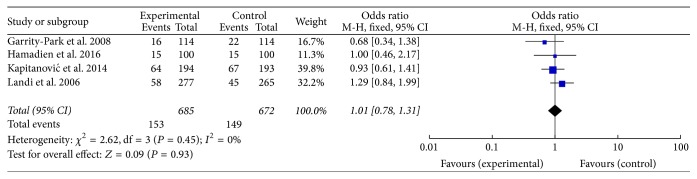
Calculated OR and 95% CIs for the associations TNF-*α* T-857C polymorphism and colorectal cancer risk in the TC versus CC model.

We included four studies to describe the association between the TNF-*α* T-857C polymorphism and colorectal cancer risk (Figure 5). However, our analysis showed no association between the polymorphism and colorectal cancer risk. The result of stratified analyses based on ethnic subgroups also revealed negative association. The sensitivity analysis showed the results without substantive change.

#### 3.2.4. Association between the TNF-*α* T-857C Polymorphism and Cervical Cancer and Prostate Cancer Risk

Of all the included studies, only three case-control studies involving 480 cases and 520 controls focused on cervical cancer and two studies involving 1336 cases and 2517 controls on prostate cancer. Meta-analysis of our result revealed negative association between the polymorphism and these two cancers. Table 2 showed the result of our analysis.

### 3.3. Publication Bias

**Figure 6:**
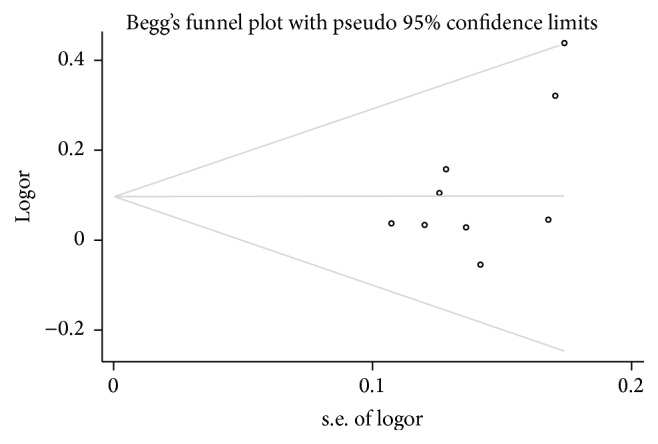
Funnel plot analysis to detect publication bias for TT + TC versus CC.

Due to limitations of the quantity of included studies, we just test the publication bias between target gene polymorphism and gastric cancer. Funnel plots were conducted to assess the publication bias, and no evidence of asymmetry was observed (Figure 6 for TT + TC versus CC model). This result was further supported by the analysis using Egger's test (T versus C: *P* = 0.06; TT versus CC: *P* = 0.14; TC versus CC: *P* = 1.00; TT versus TC + CC: *P* = 0.14; TT + TC versus CC: *P* = 0.83).

## 4. Discussion

Genetic factors have been shown to influence the susceptibility of patients to various diseases and have attracted increasing attention. Chronic inflammation and cytokines are thought to play the most important role in tumor promotion and progression by driving angiogenesis, cell metastasis, and immune-suppression. TNF is an important infectious agent in inflammation progression as well as cancer development [39]. In our meta-analysis, we aggregated data from published studies to estimate genetic associations between the TNF-*α* T-857C polymorphism and the susceptibility of five common cancers. Our analysis provided some evidence to support an elevated risk between the TNF-*α* T-857C polymorphism and gastric cancer and hepatocellular cancer susceptibility. But no associations were found in the remained three cancers (colorectal cancer, cervical cancer, and prostate cancer). In the stratified analysis by ethnicity for the TNF-*α* T-857C polymorphism and gastric cancer susceptibility, we found a significant risk in Asian populations rather than Caucasian populations, suggesting that the increased gastric risk may be ethnospecific. In the stratified analysis based on HWE, we found an elevated risk between the TNF-*α* T-857C polymorphism and hepatocellular cancer susceptibility. The results of HWE indicated that studies out of HWE might bias the result. Hence, more high quality primary studies are needed. We did not found any meaningful associations in the stratified analysis in all included cancer types by source of control and genotyping method.

To date, numerous related studies have been conducted to investigate the association between the TNF-*α* T-857C polymorphism and disease risk; however, the exact role of TNF-*α* T-857C as a carcinogen is still controversial. The previous meta-analysis by Wei et al. in 2011 also investigated the relationship between the TNF-*α* T-857C polymorphism and hepatocellular cancer susceptibility [40]. However, no association was observed between TNF-*α* T-857C polymorphism and hepatocellular cancer susceptibility in their study. Compared with their study, our meta-analysis had a lager sample size than them, which increased the statistic power. We included 4 studies, consisting of 807 patients and 1510 health controls, and revealed that TNF-*α* T-857C polymorphism is associated with a significant increased risk of HCC in homozygote model and recessive genetic model. With regard to the TNF-*α* T-857C polymorphism and gastric cancer susceptibility, the study of Cen and Wu [41] was published in 2013. Compared with the previous meta-analysis, we retained the most recent study which increased new cases for analysis when two publications [25, 42] were available. Moreover, in our meta-analysis, we conducted four subgroups analysis based on ethnicity, source of control, genotyping method, and HWE for controls and explained these roundly, while they just conducted subgroups analysis based on race. Thus, our results are more reliable and dependable. Hence, this is the most comprehensive meta-analysis that investigated the relationship between the TNF-*α* T-857C polymorphism and gastric cancer susceptibility.

It is important to note the limitations of our meta-analysis. First, heterogeneity is one of the important issues in genetic association meta-analysis. In our study, some genetic models showed clear homogeneity while others had various heterogeneities, either in total populations or in subgroup analysis. Heterogeneity may be caused by different environment or lifestyle; however, we could not study these factors due to lack of individual data. Second, it should be noted that publication bias is a potential threat to the validity of our meta-analysis because of limitations of the quantity of included studies. In addition, only articles published in English were selected and this may result in language bias leading to an overestimation of effect sizes. Therefore, the statistic power of this meta-analysis might be affected and false positive or false negative rate might occur. Third, in some cancer types, since the number of relevant original documents was limited, there was not enough power to identify the relationship between TNF-*α* T-857C variant and cancer risk. Thus, further identification based on well-designed studies with large sample sizes is needed. Fourth, as we know, cancer is a multifactorial disease and genetic mutations, environmental changes, lifestyle, diet, age, and gender may be factors in the development of cancer. Like all meta-analyses, it is a secondary retrospective study; therefore, we could not explain the fundamental underlying mechanisms clearly due to unadjusted data.

## 5. Conclusions

This meta-analysis with published data suggested that the TNF-*α* T-857C polymorphism is a risk factor for gastric cancer, especially in Asian populations. Our result also indicated that the TNF-*α* T-857C polymorphism also plays an important role in hepatocellular cancer development. There is lack of association between the TNF-*α* T-857C polymorphism and colorectal cancer, cervical cancer, prostate cancer, and breast cancer. However, considering the limited objectives of this meta-analysis, further studies providing adjusted data, large sample size, and gene-environment detailed information are needed to assess the findings.

## Figures and Tables

**Table 1:** Characteristics of published studies included in this meta-analysis.

First author	Country	Ethnicity	Cancer type	Sample size	Control source	Case^a^	Control^a^	Methods	HWE
de Oliveira 2015	Brazilian	Caucasian	Gastric cancer	262/240	HB	157/95/10	157/64/19	PCR-RFLP	<0.01
Yang 2009	Korean	Asian	Gastric cancer	84/325	PB	49/33/2	227/92/6	PCR	0.34
Hou 2007	Poland	Caucasian	Gastric cancer	304/416	PB	226/74/4	312/99/5	TaqMan	0.36
Sugimoto 2007	Japanese	Asian	Gastric cancer	105/172	HB	66/27/12	125/40/7	PCR-RFLP	0.11
Shirai 2006	Japanese	Asian	Gastric cancer	168/479	PB	102/62/4	316/146/17	TaqMan	0.98
Zambon 2005	Italian	Caucasian	Gastric cancer	129/644	HB	83/44/2	404/227/13	PCR-RFLP	<0.01
Wu 2004	Chinese	Asian	Gastric cancer	204/210	PB	148/51/5	152/56/2	PCR	0.20
Ohyama 2004	Japanese	Asian	Gastric cancer	300/472	HB	193/98/9	312/144/16	TaqMan	0.90
Lee 2004	Korean	Asian	Gastric cancer	341/261	PB	229/97/15	185/69/7	PCR	0.85
Hamadien 2016	Arabian	African	Colorectal cancer	100/100	HB	85/15/0	85/15/0	TaqMan	0.42
Kapitanović 2014	Croatian	African	Colorectal cancer	200/200	PB	130/64/6	126/67/7	TaqMan	0.60
Garrity-Park 2008	American	Caucasian	Colorectal cancer	114/114	HB	98/16/0	92/22/0	PCR	0.25
Landi 2006	Spanish	Caucasian	Colorectal cancer	281/268	HB	219/58/4	220/45/3	TaqMan	0.68
Yang 2015	Chinese	Asian	HCC	298/889	HB	203/79/16	622/228/39	PCR-RFLP	<0.01
Shin 2015	Korean	Asian	HCC	157/201	PB	116/35/6	142/53/6	PCR–RFLP	0.70
Jung 2009	Korean	Asian	HCC	227/ 365	HB	137/75/15	258/100/7	PCR	0.45
Wang 2003	Japanese	Asian	HCC	125/55	PB	80/44/1	35/19/1	PCR	0.38
Kohaar 2014	Indian	Caucasian	Cervical cancer	150/200	HB	99/44/7	102/85/13	PCR-RFLP	0.40
Nieves-Ramirez 2011	Mexican	Caucasian	Cervical cancer	191/205	PB	93/82/16	114/76/15	PCR	0.64
Deshpande 2005	Hispanic	Caucasian	Cervical cancer	139/115	HB	116/22/1	84/26/5	PCR	0.12
Kesarwani 2009	Indian	Caucasian	Prostate cancer	197/256	PB	136/57/4	196/56/4	PCR	1.00
Danforth 2008	Hispanic	Caucasian	Prostate cancer	1139/1378	HB	923/203/13	1110/254/14	TaqMan	0.90

^a^(A/B/C): A, B, and C represented the number of genotypes CC, TC, and TT, respectively; HCC, hepatocellular cancer; HWE, *P* value for Hardy-Weinberg equilibrium for TNF-*α* T-857C polymorphism among controls; PCR, polymerase chain reaction; RFLP, restriction fragment length polymorphism; TaqMan, a fluorogenic exonuclease assay.

**Table 2:** Results of overall and subgroups analyses.

	No	T versus C			TT versus CC			TC versus CC			TT versus TC + CC			TT + TC versus CC		
		OR [95% CI]	*P*(Z)	*P* ^a^	OR [95% CI]	*P*(Z)	*P* ^a^	OR [95% CI]	*P*(Z)	*P* ^a^	OR [95% CI]	*P*(Z)	*P* ^a^	OR [95% CI]	*P*(Z)	*P* ^a^
Gastric cancer																
Total	9	1.12 [1.01, 1.25]	**0.04**	0.46	1.12 [0.79, 1.58]	0.52	0.20	1.16 [1.02, 1.33]	**0.02**	0.58	1.05 [0.75, 1.48]	0.78	0.15	1.16 [1.02, 1.32]	**0.02**	0.64
HWE, yes	7	1.01 [0.70, 1.45]	0.96	<0.01	1.42 [0.95, 2.12]	0.08	0.42	1.16 [1.00, 1.34]	0.06	0.68	1.36 [0.89, 2.07]	0.16	0.40	1.18 [1.02, 1.36]	0.02	0.60
HWE, no	2	0.99 [0.79, 1.25]	0.96	0.64	0.57 [0.28, 1.16]	0.12	0.69	1.19 [0.76, 1.85]	0.45	0.11	0.51 [0.26, 1.03]	0.06	0.56	1.10 [0.81, 1.48]	0.54	0.27
Caucasian	3	1.01 [0.84, 1.21]	0.94	0.88	0.66 [0.35, 1.22]	0.18	0.63	1.13 [0.91, 1.40]	0.28	0.23	0.60 [0.33, 1.12]	0.11	0.52	1.07 [0.87, 1.32]	0.50	0.52
Asian	6	1.19 [1.04, 1.37]	**0.01**	0.38	1.46 [0.96, 2.22]	0.08	0.32	1.18 [1.00, 1.40]	**0.04**	0.63	1.38 [0.91, 2.09]	0.13	0.29	1.21 [1.03, 1.42]	**0.02**	0.56
HB	4	1.09 [0.93, 1.28]	0.28	0.15	1.04 [0.46, 2.34]	0.93	0.04	1.17 [0.96, 1.42]	0.12	0.42	0.98 [0.42, 2.27]	0.96	0.03	1.14 [0.95, 1.38]	0.16	0.41
PB	5	1.15 [0.99, 1.34]	0.06	0.71	1.34 [0.77, 2.32]	0.30	0.71	1.16 [0.97, 1.38]	0.10	0.44	1.26 [0.73, 2.18]	0.40	0.65	1.17 [0.99, 1.39]	0.07	0.54
PCR	3	1.22 [0.99, 1.50]	0.06	0.50	1.82 [0.89, 3.75]	0.10	0.90	1.17 [0.91, 1.49]	0.22	0.24	1.73 [0.84, 3.54]	0.13	0.83	1.21 [0.96, 1.54]	0.11	0.31
PCR-RFLP	3	1.16 [0.83, 1.61]	0.38	0.08	1.10 [0.32, 3.77]	0.88	0.02	1.21 [0.94, 1.55]	0.13	0.27	1.03 [0.29, 3.67]	0.97	0.01	1.18 [0.93, 1.50]	0.16	0.26
TaqMan	3	1.07 [0.90, 1.26]	0.44	0.89	0.89 [0.49, 1.61]	0.70	0.89	1.13 [0.93, 1.38]	0.22	0.63	0.85 [0.47, 1.53]	0.59	0.84	1.11 [0.92, 1.35]	0.28	0.73
HCC																
Total	4	1.16 [0.89, 1.51]	0.27	0.08	1.65 [1.06, 2.57]	**0.03**	0.14	1.10 [0.90, 1.34]	0.35	0.33	1.61 [1.04, 2.49]	**0.03**	0.19	1.16 [0.96, 1.40]	0.13	0.16
HWE, yes	3	1.16 [0.77, 1.77]	0.48	0.05	2.29 [1.16, 4.51]	**0.02**	0.14	1.13 [0.87, 1.48]	0.37	0.19	2.21 [1.12, 4.34]	**0.02**	0.20	1.22 [0.94, 1.57]	0.13	0.09
HB	2	1.32 [0.90, 1.94]	0.15	0.04	2.13 [0.68, 6.67]	0.19	0.04	1.19 [0.95, 1.50]	0.14	0.24	2.00 [0.70, 5.69]	0.20	0.05	1.30 [0.90, 1.87]	0.16	0.10
PB	2	0.93 [0.66, 1.29]	0.65	0.90	1.06 [0.36, 3.09]	0.92	0.51	0.87 [0.59, 1.30]	0.51	0, 59	2.00 [0.70, 5.69]	0.86	0.48	0.89 [0.61, 1.31]	0.56	0.72
PCR	2	1.08 [0.86, 1.35]	0.51	0.66	1.20 [0.66, 2.17]	0.55	0.47	1.05 [0.80, 1.39]	0.71	0.90	1.18 [0.66, 2.12]	0.58	0.47	1.07 [0.83, 1.39]	0.60	0.78
PCR-RFLP	2	1.24 [0.70, 2.18]	0.46	0.02	2.35 [0.73, 7.51]	0.15	0.11	1.10 [0.64, 1.89]	0.74	0.07	2.45 [1.22, 4.93]	0.01	0.17	1.18 [0.65, 2.18]	0.58	0.04
Colorectal cancer																
Total	4	1.01 [0.80, 1.27]	0.94	0.45	0.99 [0.40, 2.41]	0.97	0.62	1.01 [0.78, 1.31]	0.93	0.45	1.01 [0.41, 2.45]	0.99	0.69	1.01 [0.78, 1.30]	0.93	0.43
African	2	0.94 [0.68, 1.29]	0.69	0.85	0.83 [0.27, 2.54]	0.75	NA	0.94 [0.65, 1.36]	0.75	0.86	0.88 [0.29, 2.67]	0.82	NA	0.93 [0.65, 1.34]	0.71	0.85
Caucasian	2	1.01 [0.58, 1.76]	0.97	0.14	1.34 [0.30, 6.05]	0.70	NA	1.00 [0.54, 1.85]	1.00	0.13	1.29 [0.29, 5.84]	0.74	NA	1.00 [0.54, 1.86]	0.99	0.12
HB	3	1.06 [0.76, 1.48]	0.72	0.33	1.34 [0.30, 6.05]	0.70	NA	1.07 [0.77, 1.49]	0.69	0.31	1.29 [0.29, 5.84]	0.74	NA	1.08 [0.78, 1.49]	0.65	0.30
PB	1	0.92 [0.65, 1.31]	0.66	NA	0.83 [0.27, 2.54]	0.75	NA	0.93 [0.61, 1.41]	0.72	NA	0.88 [0.29, 2.67]	0.82	NA	0.92 [0.61, 1.38]	0.68	NA
PCR	1	0.71 [0.36, 1.38]	0.31	NA	NA	NA	NA	0.68 [0.34, 1.38]	0.29	NA	NA	NA	NA	0.68 [0.34, 1.38]	0.29	NA
TaqMan	3	1.06 [0.83, 1.35]	0.65	0.49	0.99 [0.40, 2.41]	0.97	0.62	1.08 [0.81, 1.43]	0.60	0.54	1.01 [0.41, 2.45]	0.99	0.69	1.07 [0.82, 1.41]	0.61	0.50
Cervical cancer																
Total	3	0.75 [0.44, 1.29]	0.30	0.003	0.59 [0.23, 1.54]	0.28	0.11	0.77 [0.42, 1.42]	0.40	0.01	0.81 [0.47, 1.39]	0.44	0.20	0.75 [0.40, 1.40]	0.36	0.005
Prostate cancer																
Total	2	1.05 [0.89, 1.24]	0.57	0.11	1.20 [0.62, 2.35]	0.59	0.74	1.04 [0.86, 1.25]	0.68	0.08	1.16 [0.60, 2.27]	0.66	0.85	1.05 [0.87, 1.25]	0.62	0.08

*P* ^a^ was derived from Chi-square statistics.
